# Stress transmission towards the nucleus of the cell

**DOI:** 10.3389/fcell.2026.1790136

**Published:** 2026-03-24

**Authors:** Yiwen Zhan, Shuai Shao, Bo Liu, Xiaohui Yu

**Affiliations:** 1 Women and children’s Hospital of Dalian University of Technology, Dalian, China; 2 Faculty of Medicine, Liaoning Key Lab of Integrated Circuit and Biomedical Electronic System, Dalian, China

**Keywords:** nuclear mechanotransduction, LINC complex, nuclear lamina mechanics, chromatin remodeling, mechanosensitive gene regulation

## Abstract

Cells constantly experience mechanical forces from their microenvironment, positioning the nucleus as a central integrator of physical cues and gene regulatory programs. This review examines current evidence on how mechanical signals are transmitted from the extracellular matrix to the nucleus and how key nuclear structures respond in a context-dependent manner. The perinuclear cytoskeletal components—such as the actin cap, microtubules, and the Ca^2+^-INF2 signaling axis—are discussed as key transducers that regulate nuclear morphology and facilitate mechanosensitive nucleocytoplasmic transport. The linker of nucleoskeleton and cytoskeleton (LINC) complex is highlighted as a major conduit for conveying cytoskeletal forces across the nuclear envelope. Within the nucleus, the nuclear pore complex exhibits mechanoresponsive behavior that may modulate molecular flux and contribute to structural resilience. The nuclear lamina acts as a load-bearing scaffold associated with nuclear stiffness regulation and chromatin organization. Chromatin itself undergoes force-associated structural and epigenetic remodeling, and mechanosensitive transcription factors—including, but not limited to, Yes-associated protein and transcriptional co-activator with PDZ-binding motif (YAP/TAZ)—have been implicated in linking mechanically altered nuclear states to gene expression responses. Advances in high-resolution imaging and novel force-probing technologies are further illuminating the dynamics of nuclear mechanics. Together, current findings outline an evolving framework for understanding how extracellular mechanics interface with nuclear structure and gene regulation in health and disease.

## 1 Introduction

Cells are continuously exposed to mechanical signals from their surrounding microenvironment. The ability to sense and interpret these mechanical cues, known as mechanotransduction, plays a crucial role in development, tissue homeostasis, and the progression of diseases such as cancer and fibrosis. Rather than passively bearing external loads, cells actively generate mechanical inputs primarily through the integrin–focal adhesion (FA)–actin cytoskeleton axis, a bidirectional force-transmission pathway that conveys extracellular forces toward the nucleus and is associated with downstream signaling responses (Kalli et al., 2023; Mannion et al., 2024). This process is exemplified in pathologies like solid tumor progression, where increased stromal cell density and contractility compress the extracellular matrix (ECM), exposing cancer cells to complex mechanical stresses that can drive malignant phenotypes (Mierke, 2019; Saraswathibhatla et al., 2023; Walther et al., 2023; Dos Santos and Toseland, 2021).

**FIGURE 1:**
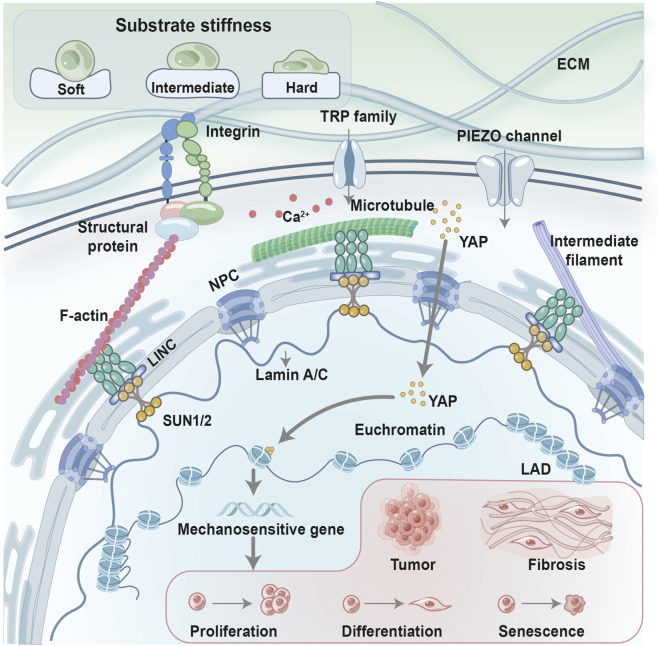
Conceptual overview of nuclear mechanotransduction. Schematic model summarizing reported mechanisms by which ECM stiffness and mechanosensitive pathways influence nuclear structure and gene regulation. Mechanical inputs are transmitted through integrins and cytoskeletal networks to the LINC complex, NPC, nuclear lamina, and chromatin, contributing to context-dependent transcriptional responses. This illustration represents an integrative conceptual framework based on multiple studies rather than a single experimental dataset. Inspired by (Lee et al., 2025).

The nucleus, the largest and stiffest organelle in the cell, serves as the central hub for integrating and responding to these mechanical stresses. It functions not merely as a genetic repository but as a mechanically responsive structure capable of integrating physical inputs with gene regulatory processes. Mechanical inputs transmitted via perinuclear actin, nuclear membrane proteins, the nuclear pore complex (NPC), and the nuclear lamina have been associated with changes in nuclear morphology and chromatin accessibility in defined experimental systems (Fu et al., 2023). External stretch, compression, or shear forces applied to the cytoskeleton can deform the nuclear envelope and are associated with chromatin reorganization and altered transcriptional outputs (Kalli et al., 2023; Saraswathibhatla et al., 2023). To maintain functionality under persistent stress, the nucleus employs dynamic adaptation mechanisms, modulating its stiffness and shape to ensure appropriate force transmission and protect genomic integrity (Liu et al., 2022). For example, Sun2-mediated nuclear softening has been shown to mitigate mechanical-stress-induced senescence (Yue et al., 2023). Such “nuclear adaptation” mechanisms enable cells to remain functional under chronic mechanical load (Nava et al., 2020). Concurrently, mechanosensitive transcriptional regulators, exemplified by Yes-associated protein and transcriptional co-activator with PDZ-binding motif (YAP/TAZ), are thought to translate mechanically induced nuclear changes into context-dependent transcriptional responses (Aragona et al., 2013). Rather than constituting a single linear pathway, nuclear membrane plasticity, chromatin mechanics, and diverse transcriptional regulators—including YAP/TAZ—are thought to interact in context-dependent and partially overlapping networks, underscoring the nucleus’s role as an active mechanical signaling hub. Mechanical signals can propagate from the ECM to the nucleus through integrins, the cytoskeleton, and the linker of nucleoskeleton and cytoskeleton (LINC) complex, influencing gene expression and cell fate decisions (Figure 1).

Despite these advances, fundamental mechanistic uncertainties persist in nuclear mechanobiology: how do mechanical forces precisely traverse the double-layered nuclear envelope to regulate gene expression with spatial and temporal specificity? Critical challenges include understanding how forces are coupled through the LINC complex to the nuclear lamina and chromatin, and how NPC permeability is mechanically tuned to control the import of transcription factors. Addressing these questions requires both mechanistic dissection of force-transmission pathways and the development of advanced tools capable of visualizing force propagation from the cytoskeleton to the nucleus. Several recent reviews have summarized key aspects of nuclear mechanotransduction, particularly cytoskeleton–LINC coupling and YAP/TAZ-associated transcriptional responses. However, a more explicitly critical framework that connects successive nuclear checkpoints from mechanically gated NPC transport and lamina mechanics to chromatin remodeling remains less clearly established. Nuclear mechanotransduction does not operate through a single, invariant pathway but instead unfolds through multiple context-dependent and sometimes competing routes, including alternative models of NPC regulation and chromatin remodeling. Differences in the mode, magnitude, and temporal profile of mechanical inputs can bias nuclear responses toward distinct molecular processes, even when similar endpoints are measured. Mechanistic models derived from specific experimental systems should therefore be interpreted as context-bound rather than universally applicable frameworks. From this perspective, inconsistencies across studies may reflect parallel or competing nuclear mechanotransduction routes rather than experimental noise. In this review, we therefore emphasize these points of contention and outstanding questions, while placing nuclear mechanotransduction within a broader landscape of mechanoresponsive transcriptional effectors beyond any single endpoint.

Therefore, this review examines the distinct yet cooperative mechanosensory roles of key nuclear components—including perinuclear actin, the LINC complex, the NPC, and the nuclear lamina—and discusses how these structures integrate mechanical cues to regulate nuclear function. Emerging technologies that enable high-resolution probing of cytoskeleton-to-nucleus force transmission are also highlighted. By critically examining current evidence, this review aims to clarify areas of consensus, highlight competing models, and refine the conceptual framing of the nucleus as a mechanically responsive regulatory structure, where coordinated interactions between cytoskeletal and nuclear structures are proposed to couple physical forces with genomic and cellular responses.

## 2 Perinuclear cytoskeletal control of nuclear mechanotransduction

Perinuclear cytoskeletal structures function as upstream mechanical integrators that transmit external forces to the nuclear surface. This section focuses on how these perinuclear assemblies—notably the actin cap, alongside microtubules, intermediate filaments, and Ca^2+^–INF2 signaling—coordinate force transmission and how such inputs are associated with changes in nuclear mechanics, NPC permeability, and the nuclear import of mechanosensitive transcription factors.

### 2.1 Perinuclear actin cap and rapid cytoskeletal remodeling in nuclear mechanotransduction

**FIGURE 2:**
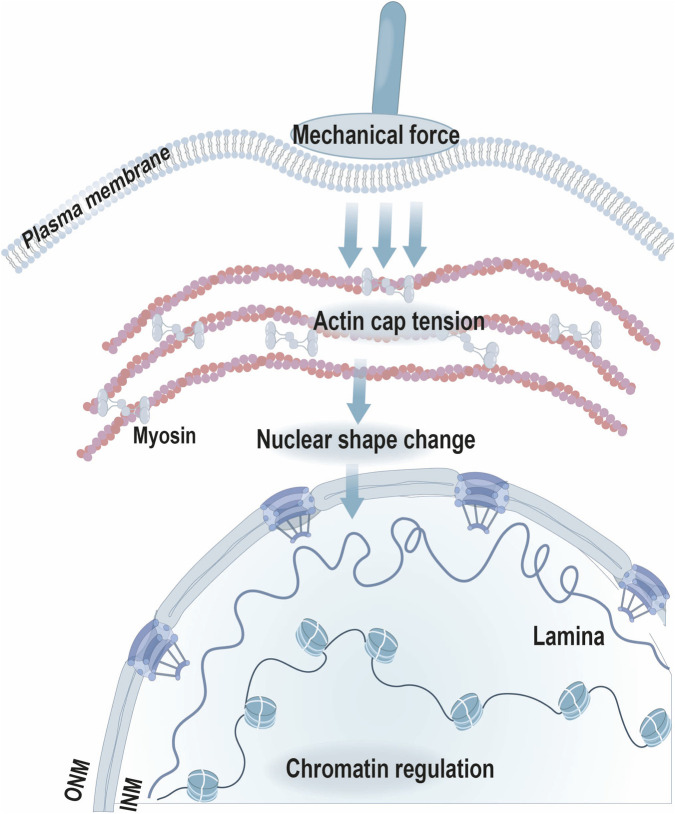
Conceptual schematic of perinuclear actin cap–mediated nuclear mechanotransduction. Schematic depiction of how actomyosin-generated tension within the perinuclear actin cap may be transmitted to the nuclear envelope through the LINC complex, contributing to nuclear shape remodeling and chromatin regulation. This illustration represents a conceptual summary of proposed mechanisms. Inspired by (Janota et al., 2020).

The perinuclear actin cap, a highly organized network of actin filaments anchored to the nuclear envelope via the LINC complex, represents a prominent mechanotransduction axis in many adherent cell types. This structure exhibits remarkable dynamic plasticity in response to force. Within minutes of mechanical stimulation (e.g., shear stress), rapid actin network assembly, directed tension redistribution, and feedback stabilization occur (Ma et al., 2024). This remodeling is accompanied by nuclear flattening and actin cap expansion, which modulate cellular mechanical properties by increasing actomyosin-generated contractility and transmitting tension to the nuclear envelope through actin-cap–LINC coupling (Figure 2). These changes in nuclear curvature have been attributed in part to actomyosin contractility, which reorganizes the perinuclear actin cap and redistributes tensile forces across the nuclear envelope via the LINC complex (Chambliss et al., 2013). The integrity of this rapid response pathway has been linked to cell-fate decisions in differentiation, senescence, and proliferation (Shiu et al., 2018; Sladitschek-Martens et al., 2022; Shi et al., 2022). Meanwhile, mechanical force-induced alterations of the actin cap reshape nuclear curvature and envelope tension, and have been associated with changes in NPC permeability and nucleocytoplasmic transport (Matsuda and Mofrad, 2022; Petrovic and Hoelz, 2022). Such changes in nucleocytoplasmic transport have been correlated with altered nuclear accumulation of multiple mechanosensitive regulators, including YAP/TAZ, MRTF/SRF, NF-κB, and β-catenin. However, whether altered transport alone is sufficient to drive downstream transcriptional reprogramming remains incompletely resolved in many systems (Ho et al., 2013; Lomakin et al., 2017; Ji, 2025).

### 2.2 Parallel modulatory pathways: microtubules and Ca^2+^–INF2 signaling

Beyond the actin cap, microtubules and intermediate filaments, particularly vimentin, also play a critical role in nuclear mechanosensitivity by aiding in force distribution and stabilizing nuclear shape under mechanical stress (Pogoda et al., 2022). Mechanical cues induce perinuclear microtubule reorganization and vimentin network strengthening, which together aid in force distribution, nuclear positioning, and the stabilization of nuclear shape, thereby subtly influencing mechanosensitive transcriptional dynamics, including YAP/TAZ as well as other regulators such as MRTF/SRF and NF-κB (Pogoda et al., 2022; Ju et al., 2024). Notably, microtubules and vimentin collaborate to form a robust mechanical framework, with microtubules helping to resist compressive forces and vimentin providing structural reinforcement, in a manner conceptually similar to the role of actomyosin in force transmission (Ju et al., 2024; Pogoda et al., 2022). This collaborative action may enhance the sensitivity of nuclear mechanotransduction under specific force regimes (Pogoda et al., 2022; Ju et al., 2024; Vanni et al., 2025; Mohammed et al., 2025). Concurrently, mechanically triggered Ca^2+^ influx activates INF2-dependent actin polymerization at the nuclear periphery. This Ca^2+^–INF2–actin axis has been reported to increase force-associated nucleocytoplasmic transport, potentially amplifying initial mechanical cues and influencing downstream transcriptional responses (Lehne et al., 2022; Kar et al., 2025). Together, these pathways operate in parallel with the core actin cap mechanism and may increase the sensitivity and temporal resolution of nuclear mechanotransduction.

### 2.3 Pathophysiological amplification in the tumor microenvironment

In pathological contexts such as solid tumors, the described mechanotransduction pathways are often upregulated or amplified, with the actin cap, microtubule networks and intermediate filaments playing critical roles in sustaining high-tension forces and promoting cellular adaptation (Maninova et al., 2017). Actin has been shown to contribute to migratory modes by coordinating protrusive activity with perinuclear force modulation (Ma et al., 2024). Actomyosin contractility has been implicated in supporting cancer-cell dissemination by generating traction forces necessary to traverse dense, crosslinked ECM (Peng et al., 2022; Antoku et al., 2023). Importantly, the Ca^2+^-dependent remodeling pathway provides a rapid “retuning” mechanism, enabling cancer cells to dynamically adjust nuclear mechanical properties in certain experimental systems to overcome transient environmental constraints (Ma et al., 2024). This pathological amplification suggests that nuclear mechanosensing pathways may constitute potential therapeutic targets. In response to mechanical stress, vimentin networks buffer compressive forces and maintain nuclear integrity, especially during cell migration and metastasis. Microtubules, on the other hand, help in force distribution across the perinuclear space, aiding in nuclear positioning and maintaining structural stability under mechanical stress (Torrino et al., 2021; Dutour-Provenzano and Etienne-Manneville, 2021). Together, vimentin and microtubules appear to reinforce each other’s mechanical functions. Such changes have been associated with increased nuclear stiffness and cellular adaptability under constrained environments, as well as enhanced invasion and survival in specific experimental models.

Together, perinuclear cytoskeletal structures function as dynamic upstream regulators that transmit and modulate mechanical inputs at the nuclear surface. Rather than operating through a single linear pathway, actin-cap–dependent tension, microtubule and intermediate filament networks, and Ca^2+^-INF2 signaling collectively shape nuclear mechanics and nucleocytoplasmic transport in a context-dependent manner. Importantly, while these pathways are closely associated with mechanosensitive transcriptional responses, the extent to which force-induced transport changes alone are sufficient to determine downstream gene programs remains an open question.

## 3 LINC complex: the core trans-nuclear-envelope force conduit

As the primary physical linkage across the nuclear envelope, the LINC complex represents a major, but not exclusive, conduit through which forces integrated by the perinuclear cytoskeleton are transmitted to the nuclear surface. Far from being a static tether, the LINC complex is a dynamic and plastic mechanosensory module that can couple cytoskeletal tension to nuclear transport and transcriptional responses in defined experimental contexts. The following sections summarize its molecular architecture, force-adaptive properties, context-dependent functions in physiology and disease, and emerging therapeutic implications.

### 3.1 Architecture and mechanical plasticity of the LINC complex

**FIGURE 3:**
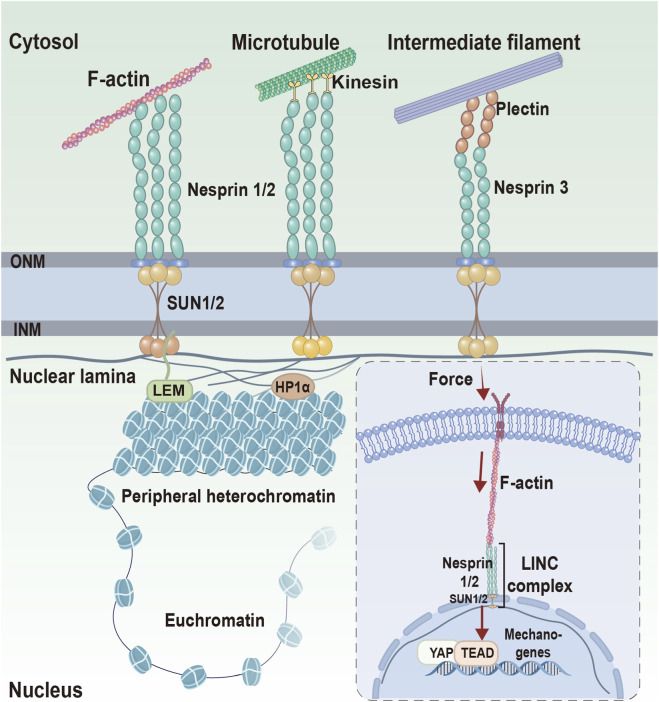
Schematic representation of the LINC complex as a structural interface linking cytoskeletal networks (actin filaments, microtubules, and intermediate filaments to the nuclear envelope through nesprin–SUN interactions. The illustration summarizes how force transmission across the LINC complex may influence nuclear lamina organization, chromatin architecture, and mechanoresponsive gene regulation. This figure represents a conceptual summary of proposed mechanisms. Inspired by (Bougaran and Bautch, 2024; Meng et al., 2024).

The core function of the LINC complex—mechanotransduction—is enabled by its dynamic molecular architecture. Spanning the inner and outer nuclear membranes, SUN proteins bind KASH-domain proteins in the perinuclear space, forming a primary physical bridge through which cytoskeletal forces can be transmitted to the nucleus (McGillivary et al., 2023). This connection has been implicated in maintaining nuclear morphology, organizing chromatin, and influencing gene expression (Zi-Yi et al., 2024). Critically, SUN–KASH interactions exhibit striking dynamic plasticity and are modulated by the cell cycle and external mechanical loads. Under high tension, enhanced SUN–KASH binding strengthens nucleoskeletal coupling and stabilizes the nuclear envelope (McGillivary et al., 2023). Structural studies show that SUN2 trimers can assemble into higher-order hexameric SUN–KASH structures that may enhance force transmission (Abdollahiyan et al., 2021). Furthermore, mechanical stress can also release autoinhibitory conformations of SUN2, increasing its force responsiveness, while proteasomal degradation of KASH proteins modulates LINC stability depending on cellular context (Young et al., 2024). Collectively, these structural and regulatory adaptations endow the LINC complex with substantial mechanical plasticity, enabling it to adapt to different mechanical environments and potentially modulate nuclear responses accordingly (Figure 3).

### 3.2 Cytoskeletal coupling diversity and force-sensitive nuclear signaling

The molecular diversity of SUN–KASH pairings enables the LINC complex to selectively couple with microtubules, actin, or intermediate filaments. Recent structural insights, such as the asymmetric 9:6 SUN1–KASH6 assembly, suggest that LINC complexes can reconfigure their architecture to match specific mechanical environments (Cruz et al., 2020; Gurusaran et al., 2024). This versatility is consistent with a role for the LINC complex in integrating mechanical and biochemical inputs. On stiff substrates, tensile forces transmitted via the integrin–LINC axis induce nuclear flattening and deformation, as demonstrated in specific experimental systems (Elosegui-Artola et al., 2017). LINC-mediated tension has been associated with changes in NPC permeability and altered nuclear localization of mechanosensitive effectors such as YAP/TAZ and MKL1, primarily in defined experimental contexts (Takata and Matsumura, 2022). Conversely, biochemical pathways such as the Hippo kinase signaling cascade (e.g., via RASSF5) can counterbalance force transmission, underscoring that LINC-dependent responses emerge from mechanochemical interplay rather than a linear cascade (Singh et al., 2024; Carley et al., 2022). In many cases, causal links between specific LINC loading states and downstream transcriptional outputs remain inferred from perturbation and localization assays rather than directly measured force transmission.

### 3.3 Cell specific modes of LINC-mediated mechanotransduction

The functional output of LINC complex activity is highly cell-type and context-dependent, shaping how mechanical cues are interpreted under physiological or pathological conditions. In stem cells (e.g., periodontal ligament stem cells), LINC-mediated force transmission has been reported to bias mechanotransduction toward osteogenic differentiation in specific stem-cell systems, with YAP nuclear localization representing one contributing regulatory axis (Meng et al., 2024). In specialized mechanosensory cells like osteocytes, LINC-dependent nucleo-cytoskeletal coupling has been shown to be necessary in certain models for sensing physiological load during bone remodeling (Birks and Uzer, 2021). Conversely, in cancer cells, this machinery is co-opted to promote malignancy. In breast cancer migration models, SUN1/2 engages in cross-talk with MKL1 to remodel the actin dynamics and enhance invasiveness (Sharma et al., 2021). Osteosarcoma cells utilize LINC to integrate inputs from both actomyosin and vimentin networks, enabling extreme nuclear deformation that facilitates migration through confined spaces (Tusamda Wakhloo et al., 2020; Birks and Uzer, 2021). This context-dependent reprogramming illustrates that the LINC complex executes tissue-specific “mechanoprograms” tailored to functional demands or hijacked in disease. Although the LINC complex is often treated as the canonical mechanical bridge across the nuclear envelope, accumulating evidence suggests that nuclear force sensing can also be supported by partially LINC-independent routes. Consistently, perturbation of upstream mechanical inputs can phenocopy aspects of the LINC mutant state, suggesting that LINC-dependent transmission is a dominant axis but not an exclusive requirement in all contexts (Wang et al., 2018).

Direct regulation of nuclear membrane tension provides one alternative route: disruption of ER–nuclear membrane continuity can unmask inner nuclear membrane tension and activate nuclear deformation–responsive signaling modules such as cPLA2, demonstrating that nuclear membrane mechanotransduction can be triggered by stress-induced remodeling of contiguous membrane systems (Shen et al., 2026). Moreover, cytoskeletal networks can also modulate nuclear mechanics through partially LINC-independent mechanisms. Intermediate filaments buffer nuclear deformation and promote mechanosensitive invasive programs in glioblastoma, while compression-induced microtubule reinforcement establishes a “mechanostat” that coordinates nuclear positioning and survival during confined migration (van Bodegraven et al., 2025; Ju et al., 2024). Thus, LINC-dependent force transmission is best viewed as a dominant but context-bounded pathway operating alongside LINC-independent nuclear membrane and alternative cytoskeletal mechanisms.

### 3.4 LINC complex in genome stability and therapeutic approaches

Beyond signal transduction, the LINC complex has been implicated in the maintenance of genomic stability. During cytokinesis, the Sun1/2–Nesprin-2 complex has been reported to sense tension on chromatin bridges and to coordinate local actin assembly via RhoA activation in defined cytokinetic contexts, contributing to the protection of chromatin integrity (Balafouti et al., 2025). LINC stability has been shown to influence cancer-cell nuclear deformability during invasion in specific cellular models. In MDA-MB-231 cells, inhibition of protein disulfide isomerase destabilizes SUN2–Nesprin-2, softens the nucleus, and enhances invasive capacity (Henretta and Lammerding, 2025). Additional regulators such as Mena modulate LINC–cytoskeleton connectivity and associated cellular responses (Li Mow Chee et al., 2023). Cytoskeletal remodeling can alter LINC load-bearing behavior, consistent with a contributory role in nuclear mechanical regulation (Wang et al., 2012; Palmer et al., 2024). These mechanistic insights have motivated growing interest in exploring LINC complex function and nuclear mechanics as potential avenues for mechanomedicine research. Emerging therapeutic strategies seek to experimentally modulate LINC conformation or nuclear envelope tension to influence diseased cellular mechanotypes. For example, nanoparticle-based delivery systems have been explored experimentally as tools to perturb nuclear membrane tension and nuclear pore permeability in controlled model systems (Yokoyama et al., 2025; Chen et al., 2025b).

Collectively, current evidence positions the LINC complex as a mechanically adaptable bridge that couples perinuclear cytoskeletal forces to nuclear architecture through dynamic SUN–KASH assemblies. Across cell types, LINC-dependent outputs reflect mechanochemical integration rather than a single linear cascade, with downstream effects shaped by cytoskeletal coupling diversity and biochemical counter-regulation. Importantly, accumulating evidence supports the view that LINC-mediated transmission is often dominant but not universally required, as nuclear force sensing can also proceed through partially LINC-independent membrane and cytoskeletal routes in specific contexts.

## 4 NPC as a force-sensitive transport gate and structural buffer

The NPC can respond to mechanical cues at the nuclear envelope by altering transport selectivity, although the underlying mechanisms and their physiological relevance are context-dependent. This section summarizes evidence that mechanical load can reshape NPC conformation, permeability, and spatial distribution, and discusses how such changes may influence nucleocytoplasmic transport, nuclear integrity, and downstream gene regulation. Cell-type variation and disease associations further highlight the NPC as a mechanically responsive structure with emerging relevance for pathophysiology.

### 4.1 From passive sieve to mechanosensitive gatekeeper

**FIGURE 4:**
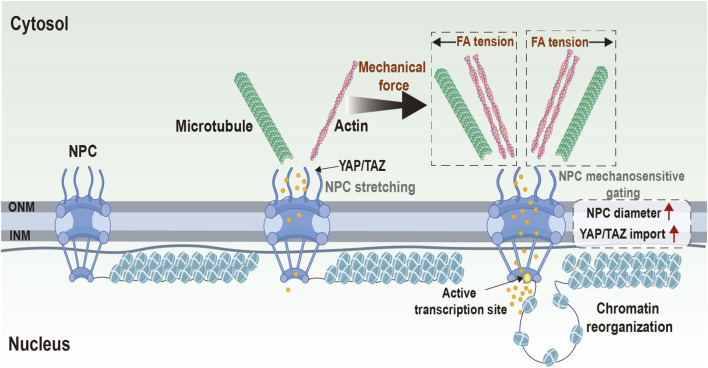
Conceptual schematic of mechanical gating at the NPC. Schematic illustration of how mechanical tension transmitted through cytoskeletal networks may be associated with alterations in NPC conformation and nucleocytoplasmic transport dynamics. Increased pore dilation has been proposed to facilitate the nuclear entry of mechanosensitive transcriptional regulators, including YAP/TAZ, potentially influencing chromatin organization and gene expression. This figure represents a conceptual summary of proposed mechanisms rather than direct experimental imaging data. Inspired by (Janota et al., 2020).

As a massive protein assembly spanning both nuclear membranes, NPC has long been viewed as a passive molecular sieve governing nucleocytoplasmic traffic. Yet accumulating evidence suggests that the NPC can respond to mechanical inputs, with changes in conformation, permeability, and spatial distribution reported in specific experimental systems (Matsuda and Mofrad, 2022). Rather than merely permitting or restricting transport, the NPC may dynamically modulate transport behavior under mechanical load. In experimental settings where nuclear stretching or envelope tension is imposed, NPC dilation and increased permeability have been reported, which may accelerate molecular transport (Petrovic and Hoelz, 2022) (Figure 4). This mechanically reversible behavior—modulated by factors such as osmotic conditions and cellular state—has been proposed as a conceptual “pressure-gating” model for how transport modes may shift between passive diffusion and carrier-mediated trafficking (Hoffmann et al., 2025; Rodríguez-Montaño Ó et al., 2025). Importantly, mechanosensitive NPC behaviors should be interpreted across distinct levels of evidence. Structural dilation of NPCs under externally applied force has been directly observed in controlled systems such as osmotic swelling (Zimmerli et al., 2021; Mosalaganti et al., 2022). Atomic force microscopy (AFM)–based nanoindentation approaches have further demonstrated that mechanical loading can remodel nuclear envelope mechanics in intact cells (Ichikawa et al., 2025). By contrast, downstream effects on transport kinetics are often inferred from permeability assays, including synthetic NPC-mimic systems, and supported by biophysical modeling (Feng et al., 2024; Matsuda and Mofrad, 2025). Two partially distinct mechanistic models have been proposed to explain force-associated changes in nucleocytoplasmic transport. The first emphasizes mechanical gating, in which cytoskeleton-transmitted tension induces pore dilation and increased permeability. The second emphasizes biochemical modulation, whereby force-dependent changes in Ran-GTP gradients or importin availability regulate nuclear transport dynamics (Scott et al., 2024). However, the extent to which such force-induced NPC dilation operates under native physiological loading conditions remains incompletely established (Lusk et al., 2025). Under typical physiological conditions, NPC dilation may be less pronounced than in experimentally imposed stretch or osmotic systems. In such contexts, the transport of transcription factors such as YAP/TAZ has been proposed to depend in part on changes in the Ran-GTP gradient or importin availability. These two mechanisms, while distinct, likely operate in a complementary manner, with NPC dilation potentially facilitating general nucleocytoplasmic transport, while Ran-GTP/importin modulation may contribute to the selective import of specific transcriptional regulators (Lusk et al., 2025). Quantitative analyses further indicate that, in the systems studied, mechanical stimulation may not increase transport uniformly; instead, it can differentially affect passive diffusion of small molecules and selective import of carrier-dependent cargoes (Andreu et al., 2022). Whether comparable NPC remodeling occurs under physiological tissue-level forces *in vivo* and directly drives transcriptional reprogramming remains speculative but biologically plausible, representing an important open question; notably, mechano-osmotic nuclear deformations have been associated with coordinated changes in nuclear shape/volume and global chromatin organization during cell-state transitions in specific experimental contexts, although the direct causal contribution of NPC dilation to these transitions remains unresolved (McCreery et al., 2025).

Although structural dilation of NPCs under defined mechanical perturbations has been directly visualized in controlled systems, its independent sufficiency to drive stable transcriptional reprogramming or cell-fate transitions remains unproven. In most systems, altered nucleocytoplasmic transport is inferred from changes in transcription factor localization or reporter dynamics rather than from experimental manipulation of pore diameter in isolation. Moreover, cell-fate outcomes typically arise from integrated signaling networks involving cytoskeletal tension, chromatin remodeling, and biochemical transport regulation operating in parallel. At present, no experimental system has demonstrated that isolated modulation of pore diameter, in the absence of parallel cytoskeletal or biochemical inputs, is sufficient to stably determine cell fate. Importantly, most transport-focused studies have used YAP as a model cargo, which may bias current mechanistic interpretation toward YAP-centered paradigms.

### 4.2 NPC structure, flexibility, and buffering role

On a structural level, the NPC has been reported to display mechanical flexibility that allows it to accommodate applied forces (Petrovic and Hoelz, 2022; Vial et al., 2023). Contrary to earlier depictions of the nuclear basket as a rigid, fishtrap-like scaffold, AFM and super-resolution imaging studies indicate that it can be highly deformable, adopting multiple conformations under mechanical load (Petrovic and Hoelz, 2022; Vial et al., 2023; Sakiyama et al., 2017). The inner ring complex has been observed to undergo reversible diameter changes between ∼40 and 60 nm, a behavior proposed to enable rapid pore adjustment in response to mechanical tension (Zimmerli et al., 2021; McCarthy and Lusk, 2022). Complementary cryo-EM analyses and computational modeling further provide indirect support for a potential protective role, in which the NPC may act as a mechanical buffer that contributes to nuclear envelope stability under mechanical stress (Vial et al., 2023). Such buffering functions are also consistent with broader studies on nuclear mechanics and intracellular stress transmission, highlighting that nuclear deformation and envelope stress responses are highly system-dependent (Kalukula et al., 2022; Lomakin et al., 2020).

### 4.3 Nucleoporins and chromatin coupling across cell types

Beyond their structural and mechanical roles, variations in NPC composition and abundance across cell types can have regulatory consequences. During cardiomyocyte maturation, for instance, a reduction in NPC number limits the nuclear entry of MAPK and other signaling molecules, which may contribute to dampening stress responses and maintaining cardiac homeostasis (Han et al., 2022). In other contexts, changes in NPC composition rather than abundance appear to play a prominent regulatory role. In *Drosophila* oocytes and epithelial cells, specific nucleoporins are involved in tethering heterochromatin and regulating gene activation or silencing, linking NPC architecture to chromatin organization and transcriptional specificity (Kotb et al., 2024). Similarly, in hematopoietic progenitors, dynamic downregulation of specific nucleoporins has been reported to influence the balance between proliferation and differentiation, underscoring the role of NPC composition in lineage-specific fate determination (Kotb et al., 2024). At the level of individual components, specific nucleoporins have been implicated in modulating nuclear responses to mechanical cues. Within this framework, Nup210 interacts with SUN2 and chromatin, linking mechanical inputs at the nuclear envelope to mechanosensitive gene expression. Loss of Nup210 disrupts heterochromatin organization and has been associated with reduced mechanically responsive gene activation, as well as altered cell adhesion and migration in breast cancer models (Amin et al., 2021). Conversely, Nup93 has been reported to limit excessive nuclear accumulation of YAP in endothelial cells, contributing to the regulation of mechanosensitive signaling. During aging or chronic inflammation, reduced Nup93 expression has been associated with sustained YAP nuclear localization and endothelial senescence, while restoration of Nup93 attenuates these responses (Nguyen et al., 2024). Notably, nucleoporin-dependent regulation may also extend to other mechanosensitive transcriptional programs beyond YAP/TAZ, reinforcing the NPC as a broader integrative hub rather than a single-pathway gatekeeper (Amin et al., 2021; Lusk et al., 2025). Together, these examples illustrate that distinct nucleoporins can differentially influence nuclear responses to mechanical cues in a context-dependent manner.

### 4.4 Disease and aging: laminopathies, HGPS, and envelope rupture

Aberrant interactions between NPC components and chromatin are increasingly recognized as drivers of tissue-specific pathology (Shin and Worman, 2022). When NPC-mediated mechanoregulation is disrupted, its consequences become particularly evident in laminopathies and aging-related diseases. In Hutchinson–Gilford progeria syndrome (HGPS), accumulation of the aberrant Lamin A isoform progerin deforms the nuclear envelope, disrupts NPC–chromatin coupling, and impairs gene regulation and DNA repair, rendering cells highly susceptible to mechanical damage. More broadly, recent findings indicate that the NPC has been proposed to contribute to nuclear stability by distributing mechanical stress across the nuclear envelope. Defects in NPC components compromise this buffering capacity, predisposing cells to nuclear envelope rupture during differentiation or under mechanical loading (Taniguchi et al., 2025). NPC composition and integrity encode cell-type-specific mechanical responses, such that their disruption leads to distinct patterns of tissue failure beyond simple transport defects.

Current evidence supports the view that the NPC functions as a mechanically responsive transport interface rather than a static molecular sieve. Structural flexibility, context-dependent modulation of permeability, and nucleoporin-specific regulatory roles collectively suggest that NPC behavior emerges from the interplay between mechanical forces and biochemical transport control. However, while mechanical gating mechanisms are supported by structural observations, their independent sufficiency to drive stable transcriptional or cell-fate outcomes remains unresolved, emphasizing the need to interpret NPC-mediated responses within broader mechanochemical networks.

## 5 Nuclear lamina as a load-bearing rheostat for nuclear mechanotransduction

Mechanical loads acting on the nucleus are borne in part by the nuclear lamina, which is positioned to couple cytoskeletal inputs with nuclear mechanics and chromatin organization. Lamin networks have been implicated in contributing to nuclear stiffness modulation, coordinating LINC-dependent force transmission, and influencing chromatin organization in defined cellular contexts through lamina–chromatin interactions. Emerging physical mechanisms highlight additional roles of the lamina in shaping nuclear mechanical behavior.

### 5.1 Architecture and force transmission through the lamina

The nuclear lamina acts as a load-bearing structure capable of transmitting mechanical inputs from the cytoskeleton to the nucleus. Lamin A/C and Lamin B form a supportive network beneath the inner nuclear membrane, contributing to the mechanical stiffness and resilience of the nucleus. Through the LINC complex, force transmission between the cytoskeleton and nuclear interior can be coordinated by the lamina, which is positioned along this pathway (Wong et al., 2022; Stephens and Miroshnikova, 2024). Mechanical inputs transmitted through LINC can deform the nuclear envelope and are associated with reorganization of the lamina and associated chromatin under external mechanical load (Danielsson et al., 2023). Notably, lamina-mediated responses are highly dependent on cell type and mechanical context.

### 5.2 A-type lamins as a mechanical rheostat for cell fate

Lamin A/C abundance scales with ECM stiffness and cytoskeletal tension, consistent with a correlation between environmental mechanics and nuclear rigidity (Lee et al., 2022; Wang et al., 2022). Changes in Lamin A/C levels have been reported to correlate with shifts in lineage bias in specific experimental systems, where higher Lamin A/C levels are associated with osteogenic differentiation and reduced Lamin A/C with adipogenic outcomes, in part through altered nuclear stiffness (Lachaize et al., 2021). However, there is limited direct causal evidence that alterations in lamin abundance alone are sufficient to determine lineage commitment across different cell types.

### 5.3 LINC–lamina coupling mechanisms and laminopathy relevance

Perturbations of A-type lamins are associated with pathological outcomes. Mutations have been reported to alter nuclear architecture and force transmission and to reduce mechanical and electrical responsiveness in cardiomyocytes, contributing to disorders such as Emery–Dreifuss muscular dystrophy (Yang et al., 2025; Yamamoto-Hino et al., 2024; Chatzifrangkeskou et al., 2023). Beyond its mechanical role, Lamin A/C has been implicated in the organization of lamina-associated domains (LADs) and is linked to lineage-specific chromatin states, suggesting a connection between nuclear mechanics and epigenetic regulation (Rønningen et al., 2015). Within this chromatin context, pathways such as the Runx2–Nesprin–Lamin axis illustrate how LINC–lamina coupling is associated with osteogenic transcriptional responses under specific mechanical conditions (Saito et al., 2025). Disruption of Lamin A/C has been reported to compromise nuclear integrity and mechanoadaptation, and is linked to nuclear envelope rupture, chromatin instability, and impaired stress responses. Such defects are implicated in multiple laminopathies, including muscular dystrophies and cardiomyopathies (Ali et al., 2025; Wang et al., 2025). In contrast, Lamin B1 primarily governs proliferation, underscoring functional divergence between Lamin subtypes. Additional lamina-associated factors, such as LAP2β, are involved in mechanoregulation by coupling lamina mechanics to chromatin remodeling (Wang et al., 2025).

### 5.4 Lamina-mediated physical constraints on chromatin organization

The nuclear lamina can impose physical and mechanical constraints that influence chromatin organization, rather than acting as a direct transcriptional endpoint. Recent studies propose that the nuclear lamina can physically constrain nuclear condensates under mechanical load, contributing to chromatin architectural stability (Zhao et al., 2022; Leite Coscarella and Kwon, 2025). Lamina-associated factors such as LAP2β have been proposed to couple this physical confinement with mechanosensitive transcription by bridging the lamina and chromatin (Sun et al., 2023). Mechanical cues originating from the ECM have been associated with changes in lamina tension and nuclear geometry, which in turn correlate with shifts in chromatin positioning and accessibility. Under stiff matrix conditions, increased lamina tension is associated with nuclear flattening and altered chromatin organization (Lee et al., 2022; Shroff et al., 2024), whereas softer environments correlate with reduced lamina tension and more relaxed chromatin configurations (Yang et al., 2025). Beyond global tension changes, mechanical forces have been reported to reposition lamina-associated domains and remodel enhancer–lamina interactions in specific experimental systems, changes that are associated with lineage-specific or pathological chromatin states, including those observed in fibrosis and cardiolaminopathies (Danielsson et al., 2023; Lee et al., 2022; Nath and Sengupta, 2025; Gacita et al., 2021).

The nuclear lamina functions as a mechanically positioned scaffold that contributes to nuclear stiffness and chromatin organization in a context-dependent manner. Variations in lamin abundance and lamina–chromatin coupling are closely associated with changes in nuclear mechanics and lineage bias, although direct causal hierarchies remain incompletely resolved. Rather than acting as a deterministic endpoint, the lamina operates within a broader mechanochemical network integrating cytoskeletal inputs with chromatin architecture.

## 6 Chromatin as a mechanically responsive component of nuclear mechanotransduction

Chromatin is not only a repository of genetic information but also a mechanically responsive nuclear material. Recent studies suggest that chromatin participates in cellular responses to the mechanical microenvironment through reversible structural and epigenetic remodeling (Niethammer, 2021). Mechanical stresses, including stretching and compression, are associated with rapid spatial reorganization of chromatin, which correlates with context-dependent changes in gene activity. Under mechanical stress, local relaxation of heterochromatin is associated with increased accessibility to transcription factors, potentially permitting transcriptional activity in regions that are otherwise condensed (Hu et al., 2024; Fujiwara et al., 2024). Together, these properties support the view that chromatin can behave as a strain-responsive component of nuclear mechanics in defined experimental contexts, rather than a purely passive substrate of force transmission.

### 6.1 Chromatin structural dynamics under mechanical load

Recent studies indicate that the heterochromatin protein HP1α can form reversible condensates via liquid–liquid phase separation (LLPS), which may increase the sensitivity of heterochromatin to mechanical perturbation (Chin et al., 2025). Under external force, partial disassembly of HP1α condensates is associated with reduced heterochromatin compaction, potentially altering the spatial accessibility of chromatin. Mechanical stretch in cardiomyocytes has been reported to reshape three-dimensional chromatin architecture and is associated with dynamic shifts in A/B compartmentalization that correlate with cardiac-specific gene expression patterns (Seelbinder et al., 2021). Similarly, in vascular endothelial cells, cyclic fluid shear stress predominantly impacts the nuclear envelope and nuclear pore interface, where chromatin repositioning is observed in association with mechanosensitive gene expression, including KLF2 (Chen et al., 2025a). Current data point to a consistent association between nuclear deformation and chromatin architectural changes at the nuclear envelope interface. However, direct causality between force application and specific architectural transitions remains incompletely resolved, emphasizing a spatial association between mechanical inputs and chromatin organization (Kotb et al., 2024).

### 6.2 Mechanically regulated epigenetic remodeling of chromatin

Beyond structural rearrangement, mechanical cues are also associated with changes in epigenetic modification states within chromatin. Mechanical stretching has been associated with chromatin decondensation and enrichment of activating epigenetic marks, which correlate with stress-responsive gene expression in specific contexts (Baranello et al., 2025). For example, mechanically induced chromatin remodeling has been associated with changes in super-enhancer–linked epigenetic landscapes, including BRD4 complexes enriched in H3K27ac (Qian et al., 2023). In parallel, the force-sensitive nuclear kinase PIP4K2B has been implicated in modulating H3K9me3 levels under mechanical stretch, which is associated with changes in chromatin compaction (Poli et al., 2023). Available evidence supports an association between dynamically reversible epigenetic states and mechanical perturbations of chromatin, which are associated with altered biochemical properties. Whether such epigenetic shifts are mechanically initiated or transcriptionally reinforced, however, remains system-dependent. Despite these advances, distinguishing direct mechanical chromatin decompaction from transcription-driven secondary remodeling remains experimentally challenging, in part due to overlapping time scales and current resolution limits (Hervé et al., 2025). Chromatin relaxation can occur within seconds following mechanical stretch, yet similar structural changes may also emerge over minutes to hours as part of transcription-coupled programs (Rodríguez-Montaño Ó et al., 2025). As a result, chromatin decompaction observed under mechanical stress may represent a primary physical response, a downstream transcriptional consequence, or a feedback interaction between both processes (Denais et al., 2016).

### 6.3 Integrative physiological and pathological consequences of chromatin mechanoremodeling

Under physiological conditions, mechanical remodeling of chromatin has been associated with adaptive transcriptional programs while contributing to nuclear mechanical homeostasis. For example, external pressure rapidly induces chromatin compaction and increases nuclear stiffness within seconds, producing a transient reinforcement effect consistent with a buffering role for chromatin under load (Tang et al., 2023). In pathological settings, however, sustained or excessive mechanical stress has been linked to maladaptive chromatin remodeling, including heterochromatin dysregulation and chromatin stiffening. For instance, fibroblasts on stiff matrices exhibit heterochromatin disinhibition and profibrotic gene activation (Jokl et al., 2023), whereas chronic overload elevates H3K9me3, restricts chromatin plasticity, and limits cardiomyocyte proliferation (Boogerd et al., 2023). Defective chromatin–lamina coupling further exacerbates nuclear deformation and genomic instability in cardiac and fibrotic disease (Jokl et al., 2023). Mechanically responsive regulatory elements have been reported to link mechanical perturbation with oncogenic transcriptional programs in specific contexts, as BRD4–H3K27ac co-occupied regulatory regions become enriched under mechanical stress (Liu et al., 2025b; Huang et al., 2021). Beyond transcriptional regulation, chromatin mechanics also contributes to genome maintenance, as DNA damage induces chromatin decondensation and nuclear softening to facilitate repair factor access (Dos Santos et al., 2021).

Mechanically perturbed chromatin does not impose a single deterministic transcriptional outcome; instead, it can bias regulatory access in ways that are interpreted by multiple mechanoresponsive pathways (Niethammer, 2021). Several regulatory systems have been implicated in translating mechanically altered chromatin states into gene expression changes, including MRTF/SRF signaling, NF-κB, β-catenin, and chromatin-associated remodeling factors (Visconti and Qiu, 2024; Guo et al., 2024; Wu et al., 2024). Among these, YAP/TAZ-dependent programs are extensively characterized in proliferative and stem-like systems (Elosegui-Artola et al., 2017; Driscoll et al., 2015). The strongest evidence for YAP/TAZ dominance derives from mesenchymal stem cells, fibroblasts, and mechanically activated tumor cells cultured on stiff matrices, where nuclear flattening and enhanced nucleocytoplasmic transport have been shown to correlate with YAP/TAZ nuclear accumulation and transcriptional activation. However, whether this apparent dominance generalizes across differentiated tissues or diverse mechanical environments remains uncertain (García-García et al., 2022; Holland et al., 2024). Notably, in cardiomyocytes and differentiated epithelial tissues, YAP/TAZ activity is often attenuated or secondary, underscoring that mechanically induced chromatin remodeling can be routed through alternative transcriptional control circuits (Seelbinder et al., 2021). Accordingly, chromatin remodeling is best viewed as a mechanically tunable context that constrains or permits transcriptional responses, rather than as a linear step that uniquely specifies a single downstream effector (Niethammer, 2021; Dupont and Wickström, 2022).

### 6.4 Parallel regulatory axes independent of YAP/TAZ

Although YAP/TAZ-dependent programs represent one extensively characterized branch of nuclear mechanotransduction, accumulating evidence indicates that mechanically induced transcriptional responses frequently proceed through partially independent regulatory axes that do not require YAP/TAZ activation. For example, MRTF/SRF signaling can be activated through actin polymerization–dependent changes in G-actin availability, linking cytoskeletal tension to transcriptional outputs without requiring YAP nuclear accumulation (Ho et al., 2013; Visconti and Qiu, 2024). Similarly, NF-κB activation has been associated with mechanically induced nuclear remodeling and chromatin accessibility changes in inflammatory and endothelial contexts (Guo et al., 2024). β-catenin signaling has also been reported to respond to matrix stiffness and cytoskeletal organization through mechanisms that are not strictly dependent on canonical YAP/TAZ activation (Wu et al., 2024). In parallel, lamina–chromatin coupling and nucleoporin-dependent chromatin tethering can modulate gene expression programs in response to mechanical inputs independently of YAP/TAZ dominance (Kotb et al., 2024; Rønningen et al., 2015). Although these pathways frequently intersect with shared cytoskeletal and nuclear mechanical processes, current data support a model in which nuclear mechanotransduction comprises multiple partially independent regulatory axes rather than a single YAP-centered cascade.

Chromatin emerges as a mechanically responsive nuclear component whose structural and epigenetic states are closely associated with changes in force exposure. While rapid chromatin reorganization can accompany nuclear deformation, distinguishing direct mechanical effects from transcription-driven reinforcement remains experimentally challenging. Rather than dictating a single deterministic outcome, mechanically perturbed chromatin appears to bias regulatory accessibility in ways interpreted by multiple partially independent signaling axes, including but not limited to YAP/TAZ-dependent programs.

## 7 Technologies enabling high-resolution mapping of force transmission to the nucleus

Recent advances in mechanical sensors and imaging technologies have expanded cellular mechanics research from two-dimensional *in vitro* systems into three-dimensional microenvironments and *in vivo* settings, improving the spatiotemporal resolution with which mechanical signals can be analyzed at the nuclear level.

### 7.1 Molecular tension sensors and genetically encoded force reporters

Among the most rapidly developing tools is the DNA microparticle tension sensor (μTS), which provides a high-throughput platform for probing the mechanical behavior of membrane receptors like T cell receptors and integrins. μTS leverages DNA-based tension gauge tethers immobilized on microparticles to establish programmable force thresholds (typically 4–56 pN), allowing force readouts on curved or three-dimensional interfaces and supporting population-level mechanical phenotyping through flow cytometry–compatible workflows (Hu et al., 2021).

Complementing interface-level force measurements, fluorescence resonance energy transfer (FRET)–based genetically encoded tension probes convert piconewton-scale mechanical extension into optical signals, enabling molecule-specific, real-time visualization of force transmission across defined receptors or intracellular proteins at subcellular resolution. In contrast to exogenous force sensing strategies such as μTS, genetically encoded FRET tension probes permit molecule-specific and spatially resolved measurements of force at defined intracellular nodes. Their core structure consists of donor-acceptor fluorescent protein modules connected by an extensible elastic peptide. Mechanical stretching of the extensible peptide reduces FRET efficiency, enabling optical readout of piconewton-scale forces acting on specific proteins (Liu et al., 2025a). *In situ* and *in vivo* extensions of FRET-based measurements further improve physiological relevance, although they remain constrained by optical scattering, motion artifacts, and challenges in spatial registration within complex tissues (Wang et al., 2023). Recently developed tunable and digital FRET tension probes allow force responses to be detected at predefined mechanical thresholds. By altering peptide mechanical stability or incorporating force-threshold modules, researchers can preset defined mechanical activation points, enabling discrete FRET responses to different force levels and shifting the readout from a “continuous gradient” to a “mechanical switch” mode (Liu et al., 2025a). Hybrid platforms combining FRET probes with DNA hairpin or electrochemical reporters enable longer-term force measurements in three-dimensional matrices and provide single-cell resolution for probing mechanical heterogeneity (Hang et al., 2021).

### 7.2 High-resolution imaging and single-molecule/structural approaches for fast mechanical events

The application of super-resolution microscopy has further advanced the spatiotemporal analysis of mechanical mechanisms. Its nanoscale resolution allows researchers to precisely observe the dynamics of cytoskeleton, focal adhesions, and membrane neck structures under force, providing insight into the physical mechanisms by which actin–geometry coupling may generate piconewton-scale push/pull forces within sub-second time scales (Nunes Vicente et al., 2023; Abella et al., 2021). Assisted by deep-learning enhanced super-resolution microscopy (SFSRM), researchers can resolve transient mechanical events on millisecond timescales, enabling analysis of rapid force-induced deformation processes (Chen et al., 2023). The deformable micro-laser force sensing technology (DEFORM) reports micro-to nanoscale mechanical perturbations in real-time within complex tissue environments, expanding the capability for deep-tissue imaging (Dalaka et al., 2024). Meanwhile, integrated strategies combining single-molecule force spectroscopy with structural biology can map force-induced conformational pathways, contributing quantitative structural and energetic information that informs the rational design of force-sensitive receptors, linker proteins, and tension probes (Erie and Weninger, 2024).

Emerging force-sensing and high-resolution imaging technologies now permit quantitative analysis of mechanical signals from the cell surface to the nucleus with increasing spatial and temporal precision. While these approaches enable visualization of force transmission and conformational dynamics at unprecedented resolution, they primarily provide correlational or structural insight rather than direct proof of long-term mechanistic causality. Continued integration of molecular force reporters with *in vivo* and temporally resolved measurements will be essential for establishing quantitative links between mechanical inputs and stable nuclear outcomes.

## 8 Outstanding mechanistic gaps and conceptual tensions

Despite major advances in nuclear mechanotransduction research, several mechanistic gaps remain unresolved. In multiple contexts, structural remodeling of nuclear components has been directly observed under defined mechanical perturbations; however, whether such changes are sufficient to drive stable transcriptional or cell-fate outcomes remains largely inferential (Kalukula et al., 2022; Lusk et al., 2025).

A central debate concerns nucleocytoplasmic transport. The “mechanical gating” model proposes that cytoskeleton-transmitted tension induces NPC dilation and increases permeability (Zimmerli et al., 2021; Mosalaganti et al., 2022). Structural dilation has been directly visualized under osmotic or imposed stretching conditions (Zimmerli et al., 2021; Ichikawa et al., 2025). However, whether comparable levels of pore dilation occur under physiological tissue-scale forces *in vivo*, and whether dilation alone is sufficient to reprogram transcriptional states, remains incompletely established (Lusk et al., 2025). In parallel, a “biochemical modulation” model emphasizes force-dependent alterations in Ran-GTP gradients or importin availability as primary regulators of nuclear transport (Scott et al., 2024). Evidence supporting this model derives largely from perturbation of transport factors and GTP metabolism rather than direct pore structural remodeling (Scott et al., 2024), highlighting that current support is mechanistically indirect but functionally consistent. These mechanisms are not mutually exclusive, and current data suggest that mechanical gating and biochemical modulation may operate in complementary or context-dependent manners (Matsuda and Mofrad, 2022; Lusk et al., 2025). Nonetheless, distinguishing which mechanism predominates under defined physiological conditions remains an important open question. Conceptually, these models differ in their primary locus of regulation: mechanical gating emphasizes structural remodeling of the pore itself as the initiating event, whereas biochemical modulation posits that force alters transport dynamics primarily through changes in molecular gradients or transport factor availability. Distinguishing these mechanisms experimentally requires temporally resolved measurements capable of separating pore structural changes from alterations in Ran-GTP or importin dynamics—an area where current methodologies remain limited.

A related and ongoing debate involves the relationship between force-induced chromatin reorganization and transcription-driven chromatin remodeling. Rapid chromatin compaction changes and nuclear stiffening responses have been reported within seconds following mechanical perturbation, consistent with a primary physical response of chromatin to strain (Tang et al., 2023; Nava et al., 2020). Conversely, chromatin relaxation and epigenetic remodeling can also emerge downstream of transcriptional activation programs triggered by mechanosensitive signaling pathways (Dupont and Wickström, 2022; Niethammer, 2021). Given that the temporal windows of these processes partially overlap and that many studies rely on endpoint chromatin accessibility or epigenetic markers, it remains experimentally unresolved whether chromatin architectural changes represent a primary mechanical deformation event or arise secondarily from transcription-coupled remodeling processes initiated upstream (Hervé et al., 2025; Rodríguez-Montaño Ó et al., 2025). Experimental paradigms capable of temporally isolating force application from transcriptional activation will be required to resolve this hierarchy.

Another unresolved question concerns the extent to which nuclear force sensing requires the LINC complex. The LINC complex is widely regarded as the canonical conduit transmitting cytoskeletal forces across the nuclear envelope (McGillivary et al., 2023; Carley et al., 2021). Moreover, perturbation of SUN–KASH interactions alters nuclear deformation and mechanosensitive gene regulation in multiple systems (Wang et al., 2018; Meng et al., 2024). However, emerging evidence indicates that nuclear membrane tension, ER–nuclear continuity, intermediate filaments, and compression-induced microtubule reinforcement can modulate nuclear mechanics through partially LINC-independent routes (Shen et al., 2026; Ju et al., 2024; van Bodegraven et al., 2025). In some experimental contexts, disruption of upstream cytoskeletal inputs phenocopies aspects of LINC perturbation, suggesting that LINC-dependent force transmission represents a dominant but not universally required axis (Wang et al., 2018). Determining when LINC acts as an obligate mechanical bridge *versus* one component within a broader mechanochemical network remains an important area for future investigation. Notably, many current conclusions are drawn from perturbation-based or correlative approaches rather than from direct force quantification at the relevant molecular nodes.

Together, these conceptual tensions highlight the need for temporally resolved, quantitative, and *in vivo* approaches to establish causal hierarchies linking nuclear mechanical inputs to long-term transcriptional reprogramming.

## 9 Conclusion and outlook

**FIGURE 5:**
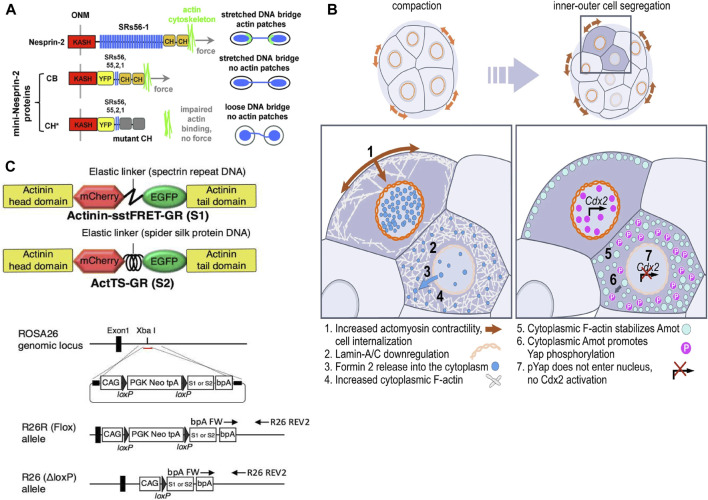
Force transmission and mechanical checkpoints at the nuclear periphery. **(A)** Domain-dependent mechanosensing by the LINC complex. Schematic depiction of Nesprin-2 mini-constructs illustrating how distinct Nesprin-2 domains differentially support force transmission and downstream actin responses under chromatin bridge–associated nuclear tension, adapted from (Balafouti et al., 2025) under CC BY 4.0 license. **(B)** Lamina-dependent organization of perinuclear cytoskeletal networks. Representative fluorescence images show altered perinuclear vimentin intermediate filament (VIF) distribution relative to the nucleus in wild-type *versus* Lamin A/C–deficient cells, illustrating coupling between lamina integrity and perinuclear force-bearing architecture, adapted from (Skory et al., 2023) under CC BY 4.0 license. **(C)** Genetically encoded FRET tension probes and *in situ*/*in vivo* implementation. Schematic showing the basic design of actinin-based FRET tension sensors (donor/acceptor fluorophores linked by an extensible element) and a reporter knock-in strategy enabling stable expression for force mapping in tissues, adapted from (Wang et al., 2023) under CC BY 4.0 license.

With the development of these tools, the nucleus is increasingly recognized as a major site of mechanical signal integration and response. A working conceptual framework has begun to emerge for understanding how external forces may be transmitted, gated, and interpreted across successive nuclear checkpoints. However, the precise contributions of each component, including the ECM, cytoskeleton, LINC complex, nuclear envelope, and chromatin, remain highly context-dependent and likely vary across cell types and force regimes (Miroshnikova and Wickström, 2022; Carley et al., 2021; Goelzer et al., 2021). Experimental studies are consistent with coordinated interactions among actin-cap–driven force transmission, LINC-dependent nuclear deformation, mechanically responsive NPC transport, and chromatin mechanical buffering at the nuclear periphery (Figure 5), although the hierarchy and sufficiency of these interactions remain incompletely defined. Importantly, key mechanistic boundaries remain actively debated, including whether NPC transport changes primarily reflect pore dilation or altered Ran/importin availability, whether chromatin remodeling represents a direct mechanical response or a secondary transcription-driven consequence, and to what extent nuclear mechanotransduction requires LINC-dependent *versus* alternative cytoskeletal routes (Shen et al., 2026; Scott et al., 2024; Rodríguez-Montaño Ó et al., 2025). On balance, advances in pathway-level understanding and enabling technologies are progressively shifting nuclear mechanobiology toward a more quantitatively testable and potentially predictive discipline (Fenelon et al., 2022). While substantial conceptual progress has been made, many mechanistic relationships remain provisional and require further quantitative validation.

This integration also provides a principled basis for emerging translational efforts in which mechanically gated nuclear states and downstream transcriptional programs may eventually be targeted more rationally. However, these strategies remain largely experimental at present, with clinical applications still in the early stages of development, rather than established clinical approaches (Fenelon et al., 2022; O'Neill et al., 2025). Moreover, nuclear mechanics interfaces with diverse transcriptional effectors beyond any single endpoint, underscoring that mechanoregulated gene programs can involve multiple context-specific regulators. Despite significant progress, key questions remain, including the precise molecular mechanisms by which mechanical signals are transmitted across the double lipid bilayer of the nuclear envelope, cell-type-specific regulatory modes of the LINC complex, and the intrinsic logic governing force-driven 3D chromatin reorganization and epigenetic modifications (Petrovic and Hoelz, 2022; Goelzer et al., 2021; Bougaran and Bautch, 2024; Lewis et al., 2024). Addressing these challenges will require deeper integration of multiscale theoretical modeling with *in situ* and *in vivo* experimental approaches to bridge molecular, cellular, and tissue-level mechanics and to establish quantitative links between force transmission and nuclear regulatory outcomes (Chen et al., 2024). Equally important will be the standardization and temporal integration of nuclear force readouts with transport gating, lamina mechanics, chromatin remodeling, and transcriptional outputs. Such integration will be necessary to distinguish rapid adaptive responses from chronic maladaptive reprogramming and to identify actionable mechanical control points within the nucleus (Petrovic and Hoelz, 2022; Bougaran and Bautch, 2024; Fenelon et al., 2022; Sohn et al., 2023). Ultimately, such integrative frameworks may advance nuclear mechanobiology toward predictive models and in the longer term inspire mechanotype-informed therapeutic concepts, although substantial work is still required to establish robust *in vivo* and clinical relevance (Chen et al., 2024; Fenelon et al., 2022).
