# Correction: Stress transmission towards the nucleus of the cell

**DOI:** 10.3389/fcell.2026.1840291

**Published:** 2026-04-24

**Authors:** Yiwen Zhan, Shuai Shao, Bo Liu, Xiaohui Yu

**Affiliations:** 1 Women and Children’s Hospital of Dalian University of Technology, Dalian, China; 2 Faculty of Medicine, Liaoning Key Lab of Integrated Circuit and Biomedical Electronic System, Dalian, Liaoning, China

**Keywords:** nuclear mechanotransduction, LINC complex, nuclear lamina mechanics, chromatin remodeling, mechanosensitive gene regulation

Affiliation Women and Children’s Hospital of Dalian University of Technology, Dalian, China was omitted for author Xiaohui Yu. This affiliation has now been added for author Xiaohui Yu.

Author Xiaohui Yu was erroneously assigned to affiliation Faculty of Medicine, Liaoning Key Lab of Integrated Circuit and Biomedical Electronic System, Dalian University of Technology, Dalian, Liaoning, China. This affiliation has now been removed for author Xiaohui Yu.

The original article has been updated.

